# Actively Targeted Nanomedicines in Breast Cancer: From Pre-Clinal Investigation to Clinic

**DOI:** 10.3390/cancers14051198

**Published:** 2022-02-25

**Authors:** Ana Isabel Fraguas-Sánchez, Irene Lozza, Ana Isabel Torres-Suárez

**Affiliations:** 1 Department of Pharmaceutics and Food Technology, Faculty of Pharmacy, Complutense University of Madrid, 28040 Madrid, Spain; aifraguas@ucm.es (A.I.F.-S.); lozza.1584043@studenti.uniroma1.it (I.L.); 2 Institute of Industrial Pharmacy, Faculty of Pharmacy, Complutense University of Madrid, 28040 Madrid, Spain

**Keywords:** active targeting, albumin nanoparticles, antibody–drug conjugates, ephrin receptors, HER-2 receptors, liposomes, nanomedicine, TNBC, trastuzumab, trop-2 glycoprotein

## Abstract

**Simple Summary:**

Despite all the efforts and advances made in the treatment of breast cancer, this pathology continues to be one of the main causes of cancer death in women, particularly triple-negative breast cancer (TNBC), and, although to a lesser degree, HER-2 receptor-positive tumors. Chemotherapy is one of the main treatments available. However, it shows numerous limitations due to its lack of selectivity. In this sense, the selective delivery of antineoplastics to cancer cells can reduce their adverse effects and increase their efficacy. The use of active targeted nanomedicine is a good strategy to achieve this selective chemotherapy. In fact, in recent decades, several active targeted nanoformulations have been approved or reached clinical investigation with excellent results. Among all nanomedicines, antibody-drug conjugates are the most promising.

**Abstract:**

Breast cancer is one of the most frequently diagnosed tumors and the second leading cause of cancer death in women worldwide. The use of nanosystems specifically targeted to tumor cells (active targeting) can be an excellent therapeutic tool to improve and optimize current chemotherapy for this type of neoplasm, since they make it possible to reduce the toxicity and, in some cases, increase the efficacy of antineoplastic drugs. Currently, there are 14 nanomedicines that have reached the clinic for the treatment of breast cancer, 4 of which are already approved (Kadcyla^®^, Enhertu^®^, Trodelvy^®^, and Abraxane^®^). Most of these nanomedicines are antibody–drug conjugates. In the case of HER-2-positive breast cancer, these conjugates (Kadcyla^®^, Enhertu^®^, Trastuzumab-duocarmycin, RC48, and HT19-MMAF) target HER-2 receptors, and incorporate maytansinoid, deruxtecan, duocarmicyn, or auristatins as antineoplastics. In TNBC these conjugates (Trodelvy^®^, Glembatumumab-Vedotin, Ladiratuzumab-vedotin, Cofetuzumab-pelidotin, and PF-06647263) are directed against various targets, in particular Trop-2 glycoprotein, NMB glycoprotein, Zinc transporter LIV-1, and Ephrin receptor-4, to achieve this selective accumulation, and include campthotecins, calicheamins, or auristatins as drugs. Apart from the antibody–drug conjugates, there are other active targeted nanosystems that have reached the clinic for the treatment of these tumors such as Abraxane^®^ and Nab-rapamicyn (albumin nanoparticles entrapping placlitaxel and rapamycin respectively) and various liposomes (MM-302, C225-ILS-Dox, and MM-310) loaded with doxorubicin or docetaxel and coated with ligands targeted to Ephrin A2, EPGF, or HER-2 receptors. In this work, all these active targeted nanomedicines are discussed, analyzing their advantages and disadvantages over conventional chemotherapy as well as the challenges involved in their lab to clinical translation. In addition, examples of formulations developed and evaluated at the preclinical level are also discussed.

## 1. Introduction

In women, breast cancer is the most frequent neoplasm and the second leading cause of cancer death. In fact, in the last decade, its incidence has increased, although, fortunate cancer-related deaths have decreased due to the improvement of early detection methods and the appearance of new treatments [1,2]. It represented 24.5% of new cancer diagnoses and 15.5% of cancer deaths in the female population worldwide in 2020 [3].

Breast tumors are divided according to the expression of hormone receptors (HR), including both estrogen (ER) and progesterone receptors (PR), and of the human epidermal growth factor receptor 2 (HER-2). In this context, breast tumors are classified into four major subtypes: (i) luminal A tumors expressing both estrogen and progesterone receptors and lacking HER-2 receptors (ER+/PR+/HER-2−), (ii) luminal B tumors that express estrogen receptors and HER-2 but lack progesterone receptors (ER+/PR−/HER-2+), (iii) HER-2 receptor-positive cancers lacking hormone receptors (ER−/PR−/HER-2+), and (iv) triple-negative breast cancer (TNBC) that lack both hormone and HER-2 receptors (ER−/PR−/HER-2−) [4,5,6]. While hormone receptor-positive tumors, especially luminal A subtype, have a low risk of developing distant metastases, the other subtypes, HER-2-positive tumors lacking ER and PR receptors and TNBC tumors, have a higher risk of developing distant metastases, which impair disease outcomes [7]. The risk is especially high in TNBC, the most aggressive and difficult to treat subtype. It shows a median overall survival rate of 1 year, much lower than the other breast cancer subtypes, with a median overall survival rate of around 5 years [4].

The treatment of breast cancer has a multidisciplinary approach that includes local treatments (surgery and radiation) and systemic therapies (chemotherapy, hormonotherapy, poly (ADP-ribose) polymerase (PARP) inhibitors, and immunotherapy such as immune checkpoint inhibitors), and depends on the cancer subtype and disease stage [8]. In metastatic breast cancer, systemic treatment, especially chemotherapy, represents the mainstay option, being usually administered preoperative (neoadjuvant) and postoperative (adjuvant), while local therapies are used with palliative properties [9].

For hormone receptor-positive tumors (luminal A and B subtypes), the first systemic therapy consists of the use of endocrine agents, such as tamoxifen, to block the hormone-mediated pathways that stimulate tumoral growth [10]. In HER-2-positive tumors, HER-2-targeted therapies such as monoclonal antibodies such as trastuzumab, or tyrosine kinase inhibitors such as lapatinib are used, usually in combination with chemotherapy [11]. In the case of TNBC, chemotherapy represents a fundamental treatment approach. This also happens in metastatic disease of HER-2-positive tumors, where chemotherapy is also an important strategy [7]. The chemotherapeutic treatments of breast cancer consist of the administration of a cocktail of antineoplastic agents that usually includes a taxane, paclitaxel (PTX), or docetaxel (DTX); an anthracycline such as doxorubicin (DOX), and a platinum agent (carboplatin or cisplatin). Cyclophosphamide (CFA) and 5-fluorouracil are also administered in some patients [4].

**Figure 1:**
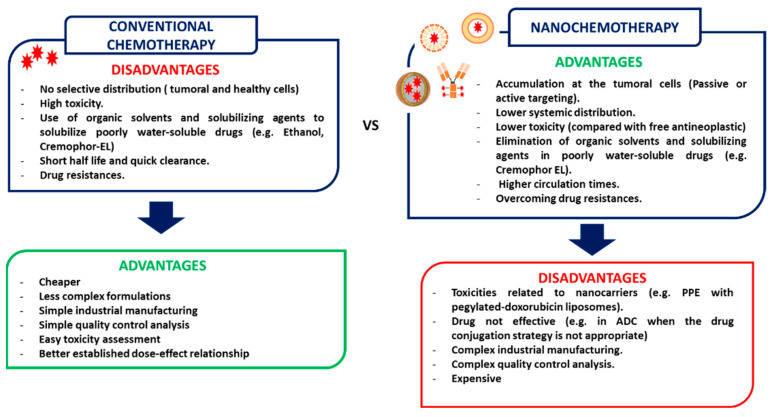
Scheme showing the drawbacks and advantages of both conventional chemotherapy and nanotechnology-based formulations of antineoplastics. PPE: palmar-plantar erythrodysesthesia, ADC: Antibody–drug conjugate.

Despite chemotherapy being a mainstay strategy in breast cancer therapy, conventional treatments have several drawbacks (Figure 1) [12]. Firstly, the distribution of antineoplastics is not selective to cancer cells, producing serious side effects that limit the administered doses of the drug. For example, the use of DOX is limited by the risk of producing cardiotoxicity, which is related to the high sensitivity of cardiomyocytes to ROS production induced by DOX. Secondly, many antineoplastics show several stability and solubility issues that challenge their use. For example, PTX is a poorly water-soluble antineoplastic that requires the use of Cremophor-EL (polyoxyethylated castor oil) and ethanol for its parenteral administration, increasing the toxicity of the formulation and limiting the administered dose, as Cre-EL is responsible for several adverse effects such as neurotoxicity, nephrotoxicity, and especially, hypersensitivity reactions. Cremophor-EL allergies are so common that the patients need to be pre-medicated with corticoids and antihistamines to prevent them [13]. Moreover, it also inhibits the binding of PTX to endothelial cells and to plasmatic albumin, which can decrease the uptake of this drug by cancer cells and increase PTX-related toxicity [14]. Thirdly, in many cases, the tumors develop drug resistance by increasing their efflux from cells, by improving DNA repair mechanisms and developing apoptosis evading/survival pathways [15]. The development of drug resistance has been related to most of the conventional antineoplastics and leads to the relapse of tumors, decreasing survival times [16].

**Figure 2:**
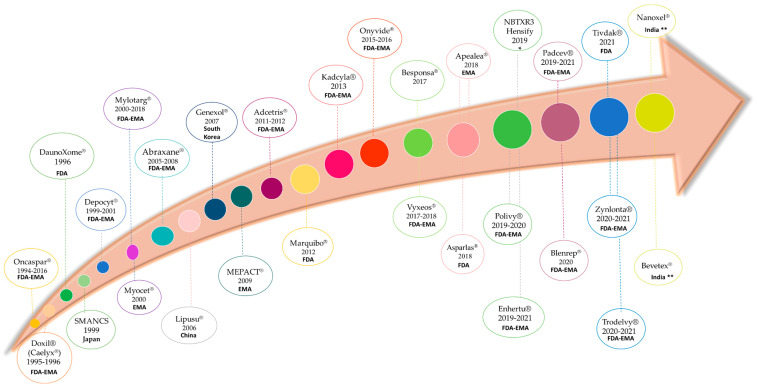
Timeline of currently approved nanomedicines for cancer. * Hafnium oxide nanoparticles that are stimulated with external radiation. Received CE-Mark. ** Approval year in India there is no available.

The use of nanotechnology-based chemotherapeutic formulations has numerous advantages over conventional chemotherapy, allowing the overcoming of these challenges. Firstly, nanomedicines (via a passive or active targeting strategy) tend to accumulate at the tumors, diminishing the systemic biodistribution of the anticancer agent, and, consequently, their related adverse effects. Secondly, they allow the administration of highly lipophilic agents, such as PTX, without using organic solvents such as ethanol or solubilizing agents as Cremophor-EL, eliminating the adverse effects related to these excipients, and, consequently, the overall toxicity of the formulation [12,17]. Thirdly, the use of nanosystems carrying antineoplastic agents can overcome drug resistances mechanisms, especially those related to the efflux pumps, as nanocarriers are not substrates of these pumps, and, consequently, the drug is retained into the cells [18,19]. Figure 2 displays nanomedicines that are currently approved for cancer.

This review is aimed at the use of active targeted nanoformulations in breast cancer chemotherapy, analyzing the currently available formulations developed as an alternative to conventional chemotherapy. This manuscript focuses on HER-2-positive and triple-negative tumors, due to the importance of chemotherapy in these neoplasms, especially when tumors have metastasized. 

## 2. Nanomedicine Based Strategies for Targeting Cancer Cells

**Figure 3:**
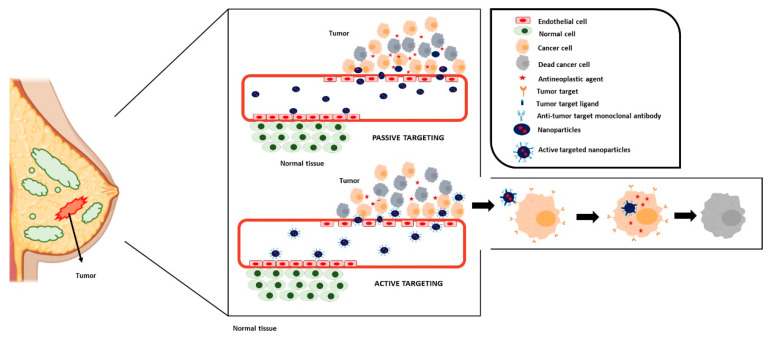
Scheme of passive and active targeting strategies. In passive targeting strategy, nanomedicines tend to accumulate at tumors due to EPR effect. In active targeting strategy, a ligand whose receptor is solely expressed or overexpressed at tumor cells is incorporated in nanomedicines. This ligand binds its receptor, and nanomedicines are selectively internalized by tumor cells.

Nanoparticles can target tumor cells due to their unique particle size characteristic through a passive targeting phenomenon. The blood vessels that line the tumors have endothelial cells with loose junctions that allow the extravasation of the nanomedicines to the tumoral microenvironment. However, in healthy tissues, excluding some specific organs such as the liver, the non-fenestrated endothelium limits the access of the nanoformulations. Moreover, tumor vasculature has low lymphatic drainage that limits the clearance of extravasated compounds. This phenomenon that is characteristic of tumors and allows the accumulation of the nanomedicines at the tumor level is known as the enhanced permeability and retention effect (EPR) [20,21] (Figure 3). In the context of solid tumors, including breast cancer, the discovery of this effect served as a key cornerstone for obtaining a selective tumor accumulation of the chemotherapeutic agents, and, consequently, a decrease in the antineoplastic-related toxicity. An increase in the antitumor efficacy may be also achieved. Many of the nanoformulations that are currently approved for cancer therapy, including breast cancer (Table 1), are based on this strategy [22,23,24]. However, nowadays, the importance of EPR in humans is controversial. In patients, nanoformulations tend to be accumulated at the tumors. However, it varies heavily between carcinomas and between patients. Recently, it has been demonstrated that the mechanism that uses the nanoformulations to enter solid tumors is much more complex than the extravasation through the gaps of tumor vasculature, and that, for example, immune cells from the tumor microenvironment are involved [25,26]. The complexity of passive targeting may explain why in most of the nanoformulations accumulated by the EPR effect, a considerable decrease in the antineoplastic toxicity is achieved, as the systemic distribution of the drug is more restricted compared to the free agent, but no significant higher efficacy is usually observed. For example, this occurs in the approved liposomal formulations of doxorubicin (Doxyl^®^/Caelyx^®^ and Myocet^®^), which show a lower cardiotoxicity risk (around 3-fold reduction) than free DOX, but a similar antitumor efficacy [27,28,29].

A higher and more specific accumulation at the tumor level is achieved using the active targeting approach [30,31]. This approach consists of using a ligand that is specifically recognized by receptors solely expressed or overexpressed at cancer cells. The active targeted nanomedicines combine both the passive accumulation due to the EPR effect and this specific accumulation due to ligand–receptor interactions. Two different targeting moieties have been used: monoclonal antibodies and small molecules such as vitamins, peptides, or carbohydrates among others [32]. In both cases, to obtain a selective accumulation of the antineoplastic agent, it can be conjugated to the targeting ligand, forming antibody–drug conjugates (ADCs) or ligand–drug conjugates, or entrapped in a nanocarrier functionalized with the targeting moiety on the surface (Figure 3).

The exploited targets depend on the cancer type. In the case of HER-2-positive breast tumors, HER-2 is the most used target as these receptors participate in the progression of these tumors. The approval of the first anti-HER-2 monoclonal antibody, named trastuzumab, at the end of the 1990s, revolutionized the treatment of this neoplasm [33]. Nowadays, other anti-HER-2 monoclonal antibodies, such as pertuzumab, have been approved and are first-line therapies for the treatment of this neoplasm [34]. Apart from HER-2 receptors, the glycoprotein non-metastatic melanoma protein b (GpNMB), epidermal growth factor receptor (EGFR), and ephrin A2 (EphA2) receptors have also been exploited, with formulations targeted to these receptors evaluated in breast cancer patients with HER-2-positive tumors. In TNBC, numerous targets have been used. In the clinic, ADCs targeting trophoblast cell-surface antigen 2 (Trop-2) and NMB glycoproteins, zinc transporter LIV-1, protein tyrosine kinase 7 (PTK7) receptor, and ephrin receptor-4 have been developed. At the preclinical level, folic acid, neuropilin-1, and CD13 receptors have also been exploited as targets.

## 3. Antibody-Drug Conjugates

**Figure 4:**
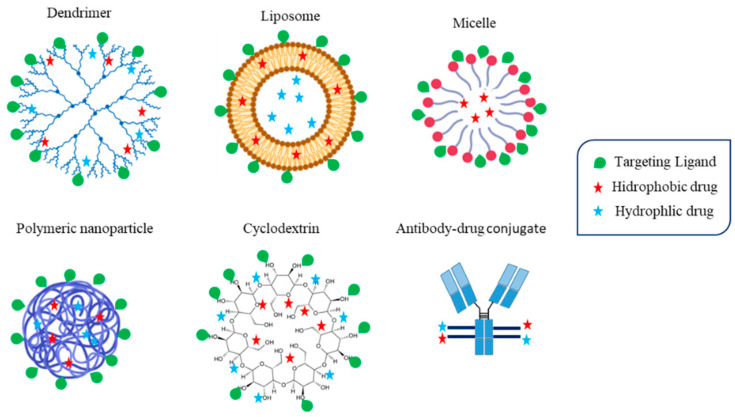
Scheme of different active targeted nanomedicines.

**Figure 5:**
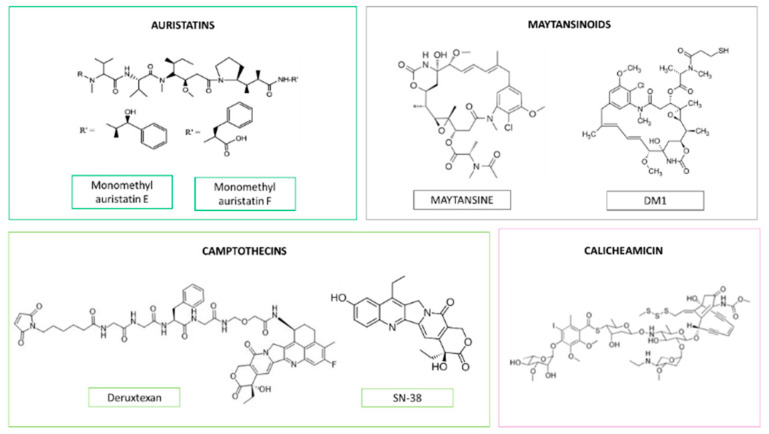
Chemical structure of most common drugs used in antibody–antineoplastic conjugates.

ADCs (Figure 4) have become a useful therapeutic tool for the treatment of cancer, with several formulations approved for the therapy of several neoplasms in the last decade, and many others under clinical research, including formulations for breast cancer therapy (Table 1) [35,36]. They consist of a monoclonal antibody, which targets a specific receptor solely expressed or overexpressed on the surface of cancer cells, bound to a potent antineoplastic agent through a cleavable or non-cleavable linker. Auristatins, maytansinoids, and calicheamicins are common drugs used in the development of ADCs [37,38] (Figure 5). The administration of these antineoplastics using ADCs allows a selective location of the drugs at the cancer cells and, consequently, a decrease in their side effects. An increase in the therapeutic efficacy, as the antineoplastic agent is delivered intracellularly at the target cell, may be also achieved. In fact, the use of this strategy allows the administration of potent but highly toxic agents that cannot be administered as free drugs. This is the case of SN-38 (Figure 5), the active metabolite of irinotecan, that shows a potent anticancer activity but serious adverse effects such as severe diarrhea, neutropenia, and myelosuppression [39,40]. ADCs containing SN-38 with a tolerable safety profile have been successfully developed. Moreover, some monoclonal antibodies exert an anticancer effect per se, such as trastuzumab, potentiating the overall tumor growth inhibition [41]. In fact, breast cancer, is one of the tumors where most antibody–drug conjugates have been developed and approved.

### 3.1. Antibody-Drug Conjugates Approved for Breast Cancer Therapy

#### 3.1.1. Kadcyla^®^

Kadcyla^®^, also named T-DM1 or Ado-Trastuzumab emtansine, was the first ADC approved by the FDA and EMA in 2013 for the treatment of solid tumors. It is formed by trastuzumab, an anti-HER-2 humanized monoclonal antibody, bound to the antimicrotubular agent DM-1 (also known as emtansine) (Figure 5) via a non-cleavage linker (*N*-succinimidyl-4-(*N*-maleimidomethyl) cyclohexane-1-carboxylate) [42]. It is used in combination with other antineoplastics for the treatment of HER-2-positive tumors, specifically for the treatment of both HER-2-positive breast and gastric tumors, being even useful in metastatic disease [43].

As aforementioned, trastuzumab exerts an antitumor activity per se. HER-2 receptors belong to the family of tyrosine kinase receptors and consist of an intracellular tyrosine kinase domain, a transmembrane region, and an extracellular domain where trastuzumab binds. This binding blocks the cleavage of the extracellular domain, inhibiting the intracellular signaling cascades mediated by this receptor (MAPK and PI3K/Akt pathways) and, consequently, increasing cell cycle arrest and suppressing cell proliferation and tumor growth. Moreover, this binding also promotes HER-2 receptor degradation and activates the antibody-dependent cell-mediated cytotoxicity [44,45].

This anticancer effect of trastuzumab makes it an excellent antibody to develop ADCs and treat HER-2-positive breast tumors. Several studies have demonstrated that at doses of 3.6 mg/kg every 3 weeks, T-DM1 was more effective and showed a lower incidence of adverse events than conventional chemotherapy in patients with metastatic or inoperable locally advanced tumors previously treated with trastuzumab and paclitaxel [46,47]. For example, Kadcyla^®^-treated patients showed a higher objective response (40.3%) and median overall survival rate (around 30 months) than the administration of lapatinib (HER-2 inhibitor) at doses of 1250 mg/day plus capecitabine 1000 mg/m^2^ twice per day. Lapatinib plus capecitabine showed a response rate of 30.8% and a median overall survival of around 25 months [48,49]. A lower recurrence rate has also been detected [43]. Interestingly, Krop et al. also demonstrated that the administration of this ADC at these doses (3.6 mg/kg every 3 weeks) in patients previously treated with anthracyclines, first-line breast cancer antineoplastics, was safe, even after 1 year of use [50]. This higher efficacy and better safety profile are related to the selective location of emtansine at the HER-2-positive tumoral cells. Thus, Kadcyla^®^ is a useful treatment for HER-2-positive breast cancer, including tumors that have metastasized.

#### 3.1.2. Enhertu^®^

Enhertu^®^ is another approved ADC targeted to HER-2 receptors that consists of trastuzumab bound to deruxtecan through an enzymatically cleavable linker [51] and that has been approved in the last two years by the FDA and EMA for the treatment of unresectable metastatic HER-2-positive breast tumors [52]. Compared to other ADCs, this formulation shows a high antineoplastic-to-antibody ratio (≈8). Most of the ADCs show a ratio of around 3–4 [53]. This elevated ratio allows the administration of higher doses of the drugs. Moreover, it has the advantage of showing a bystander effect, as deruxtecan can kill not only the target cancer cell (where the ADC has been internalized) but also the surrounding or bystander cancer cells [54,55].

Enhertu^®^ at doses in the range of 0.8 to 8.0 mg/kg administered every three weeks is well tolerated, with anemia, neutropenia, and lymphopenia being the most common adverse effects. When administered at doses of 5.4 mg/kg or 6.4 mg/kg every three weeks, it was, in general, well tolerated and showed promising clinical efficacy in HER-2-positive breast cancer patients, as more than 90% of the patients exhibited a controlled disease, even those with a low HER-2 expression [56,57]. Nowadays, phase III clinical trials are ongoing to evaluate its efficacy in tumors with this profile (NCT04494425, NCT03734029). Finally, another three clinical trials are active to evaluate its efficacy in brain metastases from HER-2-positive breast tumors (NCT04420598) and in HER-2-positive breast tumors that cannot be surgically removed or that have spread (NCT03523585, NCT03529110) comparing its efficacy with conventional chemotherapy (capecitabine) plus an HER-2-targeted agent (lapatinib or trastuzumab).

#### 3.1.3. Trodelvy^®^

Trodelvy^®^, also named sacituzumab govitecan, is the first ADC approved for triple-negative metastatic breast cancer after the failure of at least two different therapies for metastatic disease [58]. It was recently approved in 2020 by the FDA and in 2021 by the EMA. Trodelvy^®^ is formed by a monoclonal antibody targeted to Trop-2 (a glycoprotein that is overexpressed in several epithelial solid tumors as TNBC [59]) named Sacituzumab and bound to SN-38 (also known as govitecan) [60] through a pH-sensitive linker. Like Enhertu^®^, this formulation shows a high drug:antibody ratio (≈8) [61].

At doses of 10 mg/kg administered twice every 3 weeks, Trodelvy^®^ showed clinical efficacy in around 45.5% of patients with advanced metastatic breast cancer, and a median overall survival rate of around 13 months [62] without producing severe adverse effects. Gastrointestinal disorders (nausea and diarrhea), fatigue, anemia, and neutropenia were the most common side effects [63]. Compared to conventional chemotherapeutics (eribulin, capecitabine, vinorelbine, or gemcitabine), Trodelvy^®^ demonstrated a higher clinical efficacy in this kind of breast cancer, with an at least 2-fold higher objective response rate (ORR) (≥22% and ≤11%, respectively). The ORR was correlated with the expression of Trop-2 receptors, showing an ORR of around 22, 38, and 44% in tumors with a low, medium, and high Trop-2 expression, respectively [64]. Longer progression-free survival rates were also appreciated in Trodelvy^®^-treated patients [65]. A clinical benefit compared to conventional chemotherapy was also detected in HER-2-positive metastatic breast tumors, with similar objective response rates (≈32%) [66,67].

Currently, there are ongoing clinical trials to evaluate the efficacy of Trodelvy^®^ in combination with prembolizumab, an immune checkpoint inhibitor targeted to programmed cell death-1 (PD-1) protein, (NCT04468061); talazoparib, a PARP inhibitor (NCT04039230); and several antineoplastics drugs such as gemcitabine, capecitabine and albumin-conjugated paclitaxel (NCT03424005) in patients with metastatic TNBC, and with eribulin, capecitabine, gemcitabine, and vinorelbine in patients with HER-2-positive or negative mammary tumors (NCT03901339).

### 3.2. Antibody-Drug Conjugates under Clinical Research for Breast Cancer Therapy

Table 2 displays the ADCs under clinical research for breast cancer.

#### 3.2.1. Anti HER-2 Receptors ADCs

Trastuzumab-duocarmycin is an ADC formed by trastuzumab bound to a duocarmycin derivate through a protease cleavable linker [68,69]. Compared to other ADCs containing trastuzumab, trastuzumab-duocarmycin has a low drug-to-antibody ratio (≈2.8), which could limit its efficacy [70]. However, a study carried out in patients with HER-2-positive tumors demonstrated that this formulation exhibited a manageable safety profile at doses lower than 1.8 mg/kg once every three weeks, and a moderate clinical effect, with a progression-free survival of around 9 months and an overall response rate of 33% [71]. Gastrointestinal disorders (nausea and vomiting), fatigue, and conjunctivitis were the most frequent adverse effects [72]. Moreover, it has also been demonstrated to be effective in patients with Kadycla^®^-resistant tumors, which can be related to the cytotoxic payload (duocarmizyne vs emtansine) and the linker chemistry (protease-cleavable vs non-cleavable linker) [73], since, in ADCs with non-cleavable linkers, drug release is triggered by antibody metabolization so that the residues of the linker and amino acids can remain attached to the antineoplastic and interfere with its anticancer effect. In this sense, it would be better to use cleavable linkers. Currently, a phase III clinical study is ongoing in patients with advanced or metastatic HER-2-positive breast cancer previously treated with Kadcyla^®^ (NCT03262935).

Apart from trastuzumab, other anti-HER-2 monoclonal antibodies have been used as targeting entities, such as hertuzumab or HT-19. Hertuzumab has been conjugated with auristatin E via a protease cleavable linker [74], showing a promising clinical effect in patients diagnosed with HER-2-positive breast cancer and previously treated with trastuzumab, with an overall response rate above 70% [75]. In terms of toxicity, Hertuzumab-auristatin E was well tolerated up to doses of 2.5 mg/kg, with neutropenia, leucopenia, and fatigue being the adverse effects detected in approximately 50% of the treated patients. HT-19 has also been conjugated with an auristatin derivate. However, in this case, dolaflexin, a hydrophilic polymer [76], was used as a linker. This conjugation strategy allowed a very high drug-to-antibody ratio (≈12) [77], being a promising linker to the development of ADCs. Administered at doses in the range of 16–21.3 mg/kg, it was well tolerated, with headaches, gastrointestinal effects, anemia, and increased liver enzymes being common adverse effects detected at these doses. This formulation allowed the control of the disease in around 83% of the treated HER-2-positive breast cancer patients [74]. Despite all these promising results, the company decided to discontinue this formulation due to the competitive marketing of HER-2-targeted therapies [78].

#### 3.2.2. ADCs Targeted to Other Receptors

Glembatumumab-Vedotin is an ADC consisting of Glembatumumab, a monoclonal antibody targeted to the extracellular domain of the NMB glycoprotein, which is overexpressed in several tumors including breast cancer [79,80], conjugated to monomethyl auristatin E (MMAE, vedotin) [76]. While in cancer cells, NMB glycoprotein is expressed on the surface; in healthy cells, it is found intracellularly. Therefore, this formulation is almost exclusively internalized by cancer cells. However, a study undertaken in patients with recurrent breast cancer expressing NMB glycoprotein (including both HER-2-positive tumors and TNBC), showed that this antibody had similar efficacy to conventional chemotherapy, with low objectives response rates of around 6 and 7%, respectively [81]. However, when only the results of the TNBC group were analyzed, better response rates (18% and 0% in ADC and conventional chemotherapy-treated patients, respectively) were detected. These results can be attributed to the expression of NMB glycoprotein, which is much higher on the surface of TNBC cancer cells than on HER-2-positive ones [82], suggesting the potential use of glembatumumab-vedotin for TNBC therapy. A phase II clinical study has also evaluated the efficacy in this group (NCT01997333). However, results have not yet been published. It is worth mentioning that toxicity-related inconveniences were also detected, including severe peripheral sensory neuropathy, rash, renal failure, and dermatologic bullae, which led to the discontinuation of the treatment [83]. Due to this severe toxicity and the relatively poor response detected in previous studies, without any significant advance for breast cancer treatment, the investigation of this formulation has been discontinued [78].

Monomethyl auristatin E has also been conjugated with ladiratuzumab, a monoclonal antibody targeting the zinc transporter LIV-1 that is expressed in breast tumors [84], and whose expression has been linked to epidermal-to-mesenchymal transition (EMT), and, consequently, to a poor prognosis and metastases [85]. Ladiratuzumab-vedotin, administered as monotherapy at doses in the range of 0.5–2.8 mg/kg every 3 weeks, is being evaluated in patients with metastatic breast cancer (NCT01969643) [86]. Preliminary results of this study demonstrated that at these doses this formulation is, in general, well tolerated. Fatigue, nausea, alopecia, peripheral neuropathy, decreased appetite, and constipation were common adverse effects detected in more than 30% of the patients. However, most of them were mild to moderate in severity. Notwithstanding, 2 of the 69 treated patients, whose total dose exceeded 200 mg per cycle, experienced febrile neutropenia, and one of them died due to the treatment. Regarding the efficacy, an objective response rate of 32% and a clinical benefit rate (CBR) of 36% were detected [87]. Another clinical trial is evaluating the efficacy of ladiratuzumab-vedotin and pembrolizumab at doses of 2.5 mg/kg and 200 mg every three weeks in patients with unresectable locally advanced or triple-negative breast cancer (NCT03310957) [88]. A promising ORR of around 54% has been detected in triple-negative breast cancer patients after, at least, 3 months of follow-up [89]. 

Cofetuzumab-pelidotin (PF-06647020) is another ADC formulation consisting of Aur0101, an auristatin derivate, bound through a protease cleavable linker to a monoclonal antibody targeted to the PTK7 receptor [90]. This formulation is being evaluated in patients with solid tumors, including women with TNBC who received at least four previous treatments without response (NCT02222922). Preliminary results of this trial have shown a well-tolerated safety profile, with gastrointestinal disorders, alopecia, fatigue, headache, and neutropenia being the most frequent adverse effects. A promising clinical efficacy was also detected. In the case of breast cancer patients, a disease control rate of 48% was achieved when this nanoformulation was administered at doses of 2.1 and 2.8 mg/kg every 3 weeks [91]. Due to these promising results in patients with advanced or metastatic triple-negative breast tumors, a phase I study (NCT03243331) is ongoing to evaluate its efficacy in combination with Gedatolisib, a dual inhibitor of the phosphatidylinositol-3-kinase and mammalian target of rapamycin signaling pathways [92].

Finally, PF-06647263 is an antibody–drug conjugate consisting of an anti-ephrin receptor-4 monoclonal antibody conjugated with calicheamicin using a hydrazone cleavable linker [93]. This formulation has been evaluated in patients with solid tumors, including triple-negative breast cancer. In terms of toxicity, it showed an acceptable safety profile when administered at doses of 0.015 mg/kg/weekly. Mucosal inflammation, gastrointestinal disorders, thrombocytopenia, and fatigue were the most frequent adverse effects [94]. However, in terms of efficacy, a limited anticancer activity was detected: only 10% of the patients with TNBC experienced a partial response. Due to these results, the clinical trial was terminated [77].

### 3.3. Antibody-Drug Conjugates in Pre-Clinical Research

In HER-2-positive tumors, HER-2 receptors are the most exploited target, and several ADCs formulations have been developed in this regard, especially using trastuzumab, due to its own anticancer effect. For example, trastuzumab was conjugated with paclitaxel and deferoxamine (DFO)-chelated ^89^Zr. This formulation was efficiently internalized by HER-2-overexpressing breast cancer cells (SK-BR3 cells) and was barely taken up by HER-2-negative cells, also exerting an anticancer activity that was slightly higher than free paclitaxel. This indicates that this ADC may be a promising theragnostic tool for HER-2-positive tumors [95]. Trastuzumab–cisplatin conjugates have also been prepared, showing similar effects. While in HER-2-positive cancer cells trastuzumab–cisplatin conjugates exerted a similar anticancer activity than free cisplatin (IC_50_ around 20 µm in both cases), in HER-2-negative cells (including MDA-MB-231 that are triple-negative breast cancer cells), the trastuzumab–cisplatin conjugate was not effective (IC_50_ > 50 µm), while free cisplatin exerted a similar antiproliferative effect than in HER-2-positive cells (IC_50_ ≈ 10–20 µm). These results indicate that the conjugation of cisplatin with trastuzumab could be a good strategy to obtain a selective antitumor effect in HER-2-positive tumors [96]. The Trastuzumab epitope was also conjugated with doxorubicin, showing a higher anticancer activity, compared to free doxorubicin, not only in vitro in HER-2-positive breast cancer cells (SK-BR3 cells) but also in vivo in xenograft tumors developed in mice [97]. Finally, flexmab, a trastuzumab-derived monoclonal antibody, was also successfully conjugated with pyrrolobenzodiazepine through a protease cleavable linker, showing promising anticancer activity in HER-2-positive tumors developed in mice [98].

In the case of triple-negative breast cancer, an antibody–drug conjugate targeting programmed death-ligand 1 (PD-L1) has been developed, as PD-L1 has been found overexpressed in this tumor type. This formulation consists of atezolizumab, an anti-PD-L1 monoclonal antibody, bound to doxorubicin using a hydrazone cleavable linker. This formulation exhibited potent cytotoxic activity in triple-negative breast cancer cells and a significant release of IFN-γ. This indicates that this ADC can up-modulate T cell activation [99].

## 4. Albumin Nanoparticles

Due to its high biodegradability, lack of toxicity, immunogenicity, and easy elaboration process with a controllable particle size distribution, albumin has emerged as a potential nanocarrier of drugs, especially of highly lipophilic drugs as it improves their solubility and allows their administration without organic solvents or solubilizing agents [100,101]. This is particularly of interest in cancer disease, as albumin nanoparticles tend to be accumulated at the tumors. Albumin is catabolized by cancer cells to provide nutrition and energy to the fast-growing tumors [102]. Albumin nanoparticles accumulate at tumors not only via passive targeting strategy due to their small particle size but also by an active targeting strategy mediated by albondin (gp-60) receptors that are overexpressed on tumor endothelial blood vessels [103] and by SPARC proteins that have a high affinity for albumin and are overexpressed on the cancer cell surface and at the tumor interstitium [12]. Albumin nanoparticles bind to albondin receptors activating the caveolin-1 pathway and triggering the invagination of the endothelial cell membrane. Then, albumin nanoparticles enter the tumor interstitium and bind to SPARC proteins, releasing the entrapped drug [104,105,106]. This selective accumulation of the antineoplastics through albumin-based nanoparticles decreases their adverse effects and increases, in some cases, their anticancer efficacy.

### 4.1. Albumin Based Nanoparticles Approved for Breast Cancer Therapy: Abraxane^®^

Paclitaxel (PTX) is a potent frontline chemotherapeutic agent in breast cancer, especially in TNBC and metastatic diseases of other subtypes [107]. Due to its low aqueous solubility, the conventional PTX formulation (such as Taxol^®^) uses Cremophor-EL (527 mg/mL) and ethanol (393 mg as solubilizing agents, which increase the overall toxicity of the formulation. Cremophor-EL is especially toxic, producing nephrotoxicity, neurotoxicity, and hypersensitivity reactions [108]. The probability of trigger allergies is so high that patients are premedicated with antihistamines and corticoids to prevent them [109]. In this way, the administration of PTX using nanocarriers avoid the use of these excipients, decreasing the toxicity of the formulation, and, consequently, increasing the maximum tolerated dose of administered PTX. In fact, most nanomedicines that are currently approved for the treatment of breast cancer incorporate this compound as an antineoplastic. 

Abraxane^®^, which consists of albumin nanoparticles of around 130 nm entrapping PTX, was approved in the 2000s by the FDA and EMA for the treatment of different neoplasms, including breast cancer [110]. Currently, it is used in metastatic breast cancer after failure of combination chemotherapy and when standard treatment including an anthracycline is inadequate. Recently, it has been approved in combination with atezolizumab in PD-L1 overexpressing unresectable locally advanced tumors or metastatic TNBC [111].

Compared to conventional PTX, Abraxane^®^ shows a lower overall toxicity as demonstrated by the maximum tolerated doses (MTD) detected in both formulations (175 mg/m^2^ and 260–300 mg/m^2^, respectively) [12]. However, a study carried out in patients with metastatic breast cancer observed a higher rate of peripheral neuropathy that rapidly improved in Abraxane^®^-treated patients [112]. A better anticancer effect has also been detected, probably due to the possibility of administering higher doses, as Abraxane^®^ shows a lower MTD. For example, in HER-2-positive patients, the administration of Abraxane^®^ (125 mg/m^2^ three times every 3 weeks for 12 consecutive weeks) followed by epirubicin and cyclophosphamide (90 mg/m^2^ and 600 mg/m^2^ every 3 weeks) demonstrated better complete response rates than the administration of Taxol^®^ (90 mg/m^2^ three times every 3 weeks for 12 consecutive weeks) followed by epirubicin and cyclophosphamide at the same doses [113]. Similar results were also found in TNBC patients, where the use of Abraxane^®^ (125 mg/m^2^ once a week for 12 weeks) allowed a higher invasive-free survival rate than the use of Taxol^®^ (80 mg/m^2^ once a week for 12 weeks) [114]. The combination of Abraxane^®^ and atezolizumab, an anti-PDL-1monoclonal antibody was also evaluated in patients with TNBC. In this case, a higher progression-free survival rate (7.2 vs. 5.5 months) was detected in Abraxane^®^ plus atezolizumab compared with Abraxane^®^. However, a higher incidence of adverse effects that led to treatment discontinuation, almost two-fold higher (15.9% vs. 8.2%), was detected with this combination [115]. In fact, the sponsor decided to withdraw it after FDA consultation.

Currently, several clinical studies are ongoing to evaluate the efficacy of Abraxane^®^ in combination therapy. For example, being evaluated in TNBC is: (i) Abraxane^®^, carboplatin, and doxorubicin in combination with HLX10, a monoclonal antibody targeting PD-1 vs. Abraxane^®^, carboplatin, and doxorubicin (NCT04301739), (ii) Abraxane^®^ plus toripalimab after the administration of epirubicin and cyclophosphamide (NCT04418154), (iii) Etrumadenant plus Abraxane^®^, vs. pegylated liposomal doxorubicin, or IPI-549, a phosphoinositide-3-kinase-gamma inhibitor plus pegylated liposomal doxorubicin (NCT03719326), and (iv) Abraxane^®^ vs. SG001, an anti-PD-1 monoclonal antibody (NCT05068141). In HER-2-positive tumors, is being studied: (i) Abraxane^®^ plus pyrotinib maleate, an HER-2 inhibitor (NCT03919253, NCT04659499), (ii) Abraxane^®^ plus carboplatin and trastuzumab (NCT03907800), (iii) Abraxane^®^ plus pegylated liposomal doxorubicin and trastuzumab (NCT03994107), and (iv) Abraxane^®^ plus trastuzumab–emtansine conjugate and lapatinib (NCT02073916).

### 4.2. Albumin-Based Nanoparticles under Clinical Research for Breast Cancer Therapy

Apart from Abraxane^®^, albumin nanoparticles entrapping methotrexate and rapamycin have reached clinical investigation [116]. However, only rapamycin-albumin nanoparticles have been evaluated in breast cancer patients among other tumor types, such as cervical, bladder, ovarian, and prostate carcinomas (Table 2) [117]. A phase I clinical study undertaken in 27 patients with advanced carcinomas demonstrated a maximum tolerated dose of 100 mg/m^2^ of rapamycin-albumin nanoparticles weekly administered for 3–4 weeks. At these doses, most formulation-related adverse effects were mild to moderate in severity, with thrombocytopenia, mucositis, and fatigue being the most common. Severe anemia or severe dyspnea were detected in two patients [118]. This study only included one patient with advanced breast cancer. Another phase I study is evaluating the safety and efficacy of these rapamycin-albumin nanoparticles in patients with stage III breast tumors among other tumor types (NCT02646319). Nevertheless, the results have not yet been published.

### 4.3. Albumin Based Nanoparticles at Pre-Clinical Research

At the preclinical investigation, numerous albumin-based nanoparticle formulations have been developed for breast cancer therapy, especially in TNBC with promising results. For example, doxorubicin-entrapped albumin nanoparticles successfully inhibited the proliferation of MDA-MB-231 cells. However, while Lee et al. reported that doxorubicin-loaded albumin nanoparticles were more effective than free doxorubicin (cell death percentages of around 30% and 87% were detected) [119], Prajapati et al. demonstrated a significantly higher effect with the free drug than with albumin nanoparticles (cell death percentages of around 55% and 40%, respectively) [120]. Nanoparticles entrapping curcumin were also effective [121]. Interestingly, albumin nanoparticles were proven to be effective for the co-administration of several agents. Nanoparticles entrapping cyclopamine and doxorubicin were effective in killing these TNBC cells and reverting doxorubicin resistance, as they decrease the expression of the gP-glycoprotein [122]. Quercetin and docetaxel co-loaded albumin nanoparticles were also effective in overcoming docetaxel resistance mediated by gP-glycoprotein and demonstrated to decrease the growth of these tumors in xenograft mice models. Moreover, in vivo studies showed a higher accumulation of the antineoplastic at the tumors when administered entrapped in albumin nanoparticles than when used as a free drug due to albumin-mediated targeting. This selective distribution at the tumor level suggests that a decrease in chemotherapeutic adverse effects could be also achieved with these albumin-based formulations [123].

Formulations coated with an extra targeting ligand have also been developed, exerting, in this case, a dual targeting to breast cancer cells. Again, in HER-2-positive cancer cells, ligands targeting these receptors have been used. For example, nanoparticles entrapping curcumin and decorated with anti-HER-2 aptamers showed a significantly higher cytotoxic activity in HER-2-positive breast cancer cells (SK-BR3 cells) than the free drug. However, non-decorated nanoparticles showed a similar effect to free curcumin [124]. Trastuzumab-coated doxorubicin-entrapped nanoparticles were also effective [125]. In TNBC, a targeting strategy to the folate receptor (as the 50–80% of TNBC cells overexpress folic acid receptor α) has been used. In this way, folic acid-decorated albumin nanoparticles entrapping a natural cytotoxic agent were efficiently internalized by folate receptors overexpressing TNBC (MDA-MB-231) cells and showed a significantly higher antiproliferative activity in these tumor cells. Nevertheless, it showed a negligible internalization in cells with a low expression of folate receptors (SK-BR3 cells). In these latter cells, a higher antiproliferative effect was not achieved with decorated nanoparticles compared with the non-decorated ones [126]. Finally, albumin particles entrapping curcumin and decorated with a programmed death-ligand 1 biding peptide also demonstrated a significantly higher cytotoxic effect than non-decorated nanoparticles and free curcumin in MDA-MB-231 cells. Moreover, they restored tumor immune response due to PDL-1/PD-1 blocking [127].

## 5. Liposomes

Liposomes are spherical nanovesicles formed by an inner aqueous core surrounded by one or more hydrophobic lipid bilayers [128]. Due to this structure, they can efficiently encapsulate both hydrophobic agents (in the lipidic bilayer) and hydrophilic agents (in the inner aqueous core) (Figure 4). Amphiphilic molecules are placed in the hydrophilic–hydrophobic interface. Among all nanocarriers, liposomes are one of the most successful systems, probably due to their safety, efficiency, and relatively easy industrial manufacturing compared to other nanoplatforms [12]. They have been successfully used to administer anticancer agents, decreasing their toxicity and, in some cases, improving their efficacy, with several marketed formulations (Table 1). For example, doxorubicin-loaded liposomes are marketed for the treatment of different neoplasms, including breast cancer, showing lower cardiotoxicity than free doxorubicin. Therefore, liposomal doxorubicin is a better option than conventional doxorubicin in elderly people, since they have a higher risk of side effects, and free doxorubicin is not recommended [129], and in combination with other agents that could increase the risk of cardiotoxicity such as trastuzumab or lapatinib [130,131]. Paclitaxel- or vincristine-loaded liposomes are also approved, showing lower toxicity than conventional formulations. It should be mentioned that all approved liposomal formulations are based on a passive targeting strategy and no active targeted liposomes have yet been approved. However, several formulations encapsulating doxorubicin are under clinical research (Table 2) [132].

### 5.1. Active Targeted Liposomes under Clinical Research for Breast Cancer Therapy

#### 5.1.1. MM-302

The MM-302 formulation consists of pegylated liposomes encapsulating doxorubicin and decorated with a single chain fraction of an anti-HER-2 monoclonal antibody [133]. These were the first active targeted liposomes that reached clinical investigation. The incorporation of an HER-2-targeting ligand on the liposomal surface allows liposomes that have been extravasated into the tumoral microenvironment to be internalized by cancer cells that overexpress HER-2 receptors. In fact, a study undertaken in patients with metastatic HER-2-positive tumors demonstrated an efficient tumor accumulation of ^64^Cu-radiolabeled-MM302 liposomes [134], indicating that this targeting strategy was effective.

This formulation, administered in breast cancer patients with locally advanced or metastatic HER-2-positive tumors at doses in the range of 8–50 mg/m^2^ every 4 weeks, was well tolerated without detecting serious and dose-limiting toxicities. Fatigue, gastrointestinal disorders, mucosal inflammation, anemia, and febrile neutropenia were the most frequent adverse effects. Due to the presence of polyethylene glycol coating, this formulation reached high concentrations in the skin, and palmar-plantar erythrodysesthesia was detected in some patients. In fact, this adverse effect seems to be characteristic of pegylated-liposomal formulations of doxorubicin, as appears in Doxil/Caelyx^®^, pegylated liposomes of doxorubicin but not in Myocet^®^, non-pegylated doxorubicin liposomes and related to the small size of pegylated liposomes. Both formulations are approved for breast cancer but are based on passive targeting accumulation. Regarding the clinical efficacy of MM-302, a moderate overall response was achieved in 13% of the treated patients [135].

These immunoliposomes were also well tolerated in combination with trastuzumab (6 mg/kg) and trastuzumab (6 mg/kg) plus cyclophosphamide (450 mg/m^2^) when administered at doses of 30 mg/m^2^ every 3 weeks. It should be pointed out that this formulation binds to a different site of HER-2 receptors than trastuzumab and, therefore, their combination could be beneficial. However, a phase II clinical trial (NCT02213744) demonstrated no clinical benefit when trastuzumab was combined with MM-302 vs. trastuzumab plus conventional antineoplastics such as gemcitabine, capecitabine, or vinorelbine [132], and the study was discontinued.

#### 5.1.2. C225-ILS-Dox

This formulation consists of pegylated liposomes loaded with doxorubicin and decorated with an antigen-binding fragment of cetuximab, a monoclonal antibody targeted to EGFR [136]. Administered monthly at doses lower than 50 mg/m^2^ (the recommended maximum tolerated dose) for a maximum of 6 months, this formulation was well tolerated in patients with solid tumors. However, in this study, no breast cancer patients were included. At higher doses, severe febrile neutropenia, septicemia, and a fatal massive oral bleed were detected in some patients—toxicities that could be related to this treatment. Regarding the efficacy, a clinical effect in terms of stable disease was detected in 39% of the patients. A complete and partial response was also detected in one patient in each case. A total of 26 patients were included in the study [137].

It should be highlighted that more than 60% of the triple-negative breast tumors overexpress epidermal growth factor receptors, and a clinical trial was launched to evaluate the efficacy of this formulation in patients with this tumor type (NCT02833766). However, this trial was recently terminated without any published results.

#### 5.1.3. MM310

MM-310 is a liposomal formulation encapsulating a pro-drug of docetaxel and decorated with an antibody targeted to the ephrin A2 receptor. A phase I clinical trial (NCT03076372) has been launched to evaluate its toxicity and establish the maximum tolerated dose in patients with several solid tumors, including patients with triple-negative breast cancer [138]. However, the results have not yet been published.

### 5.2. Active Targeted Liposomes in Preclinical Research

Numerous active targeted liposomes have also been developed and studied in preclinical models of breast cancer. In HER-2-positive tumors, paclitaxel and rapamycin co-loaded liposomes decorated with trastuzumab showed a higher cytotoxic activity in SKBR3 cells and a higher tumor growth inhibition in xenograft tumor models developed in mice. While free antineoplastics and non-decorated liposomes exerted a similar tumor growth inhibition (around 43% compared to the negative control), decorated liposomes showed a significantly higher effect, with a tumor growth inhibition of around 70% [139], indicating the potential use of trastuzumab coating to increase the efficacy of liposomal paclitaxel and rapamycin. A better cytotoxic activity was also detected in JIMT1 HER-2-positive breast cancer cells with trastuzumab-coated liposomes loaded with curcumin and resveratrol [140]. Finally, doxorubicin-loaded liposomes coated with trastuzumab and OKT-3, an anti-CD3 monoclonal antibody, have also been developed, demonstrating a higher cytotoxic effect than non-coated liposomes in HER-2-positive breast cancer cells (BT474 cells). The activation of T cells against tumor cells mediated by OKT-3, as CD3 receptors are expressed on T lymphocytes, was also appreciated [141].

In the case of triple-negative breast cancer, several targets have been exploited. For example, liposomes loaded with docetaxel or paclitaxel pH-sensitive pro-drugs (designed to be active when internalized in the tumoral cells) and coated with a monoclonal antibody targeting Ephrin A2 receptors have been developed. These formulations exhibited a higher cytotoxic effect in triple-negative breast cancer cells (MDA-MB-468 and SUM149 cells) and a higher antitumor activity in vivo in xenograft tumor models developed in mice, increasing the survival rates of the mice [96]. Moreover, another study reported that the combination of Ephrin A2-targeted docetaxel-loaded liposomes and anti-PD-1 treatments was effective in TNBC-derived tumors [142]. These preclinical studies indicate that the use of ephrin A2-targeted liposomes could be a good strategy for the administration of taxanes. Finally, liposomes loaded with benzoporphyrin and coated with folic acid have also been studied. However, in this case, higher activity was not detected in targeted liposomes compared with the non-targeted ones in TNBC cells (MDA-MB-231 cells) [143], indicating that probably the aforementioned targeting strategies are better alternatives.

## 6. Polymeric Nanocarriers

Polymeric nanocarriers also represent good nanoplatforms for the selective delivery of drugs at the action site, including antineoplastics at the tumors. Like liposomes, these systems are useful for the encapsulation of both liposoluble and water-soluble drugs (Figure 4), including their combination in the same particle, which could be an advantage to obtain a synergistic effect as both drugs can reach cancer cells at the same time. They also have the advantage of the possibility of controlling particle characteristics (e.g., particle size, loading, and drug release pattern) to obtain optimal formulations by changing the elaboration technique and by modifying the different elaboration parameters [144,145,146]. However, their industrial manufacturing is relatively more difficult compared with liposomes as all the techniques are not conducive to industrial scale-up. Nevertheless, the modification of the surface of polymeric carriers is relatively easy making possible the elaboration of active targeted formulations [147,148].

### 6.1. Polymeric Micelles

Polymeric micelles represent the third nanoplatform approved as a nanocarrier of antineoplastic agents. They are especially interesting for the delivery of poorly water-soluble drugs (Figure 4), such as paclitaxel, allowing their administration in an aqueous medium without using organic solvents or solubilizing agents such as Cremophor-EL^®^. However, they have a low loading capacity, especially compared with other nanocarriers. Moreover, after their intravenous administration, they tend to dissociate promptly due to their dilution and the impossibility of maintaining a concentration higher than the critical micellar concentration. Moreover, their dissociation triggers the release of the drug. For this reason, they are probably not the ideal active targeted nanocarrier [149]. Despite these shortcomings, several polymeric micelles encapsulating antineoplastic agents have been developed, and three formulations containing paclitaxel are currently approved (Table 1) for the treatment of different neoplasms, including breast cancer. All of them are based on passive targeting and their major advantage rely on their low toxicity (higher MTD) compared with conventional formulations due to, at least in part, the elimination of Cremophor-EL for PTX administration. Other non-active targeted formulations containing other drugs such as doxorubicin and cisplatin are also under clinical trials [150]. Several active targeting formulations have been developed and studied at the pre-clinical level.

HER-2-targeted micelles containing several anticancer agents have been designed. For example, micelles prepared with d-α-tocopheryl polyethylene glycol succinate linked to a siRNA that inhibits polo-like kinase 1 (shows a pivotal role in cell mitosis) and conjugated with trastuzumab (anti-HER-2 monoclonal antibody), were loaded with docetaxel and demonstrated a significantly higher cytotoxic effect in HER-2-positive breast cancer cells (SK-BR-3 cells) than non-conjugated micelles, with IC_50_ values of around 3.2 and 0.67 ng/mL, respectively. Moreover, they were more effective than Taxotere^®^ (conventional docetaxel formulation) and showed an IC_50_ of 1.72 µg/mL [151]. A higher overall anticancer effect in SK-BR-3 cells was also obtained with trastuzumab-decorated d-α-tocopheryl polyethylene glycol succinate polymeric micelles co-loaded with docetaxel and cisplatin. While a significant decrease in cell survival compared to free cisplatin was appreciated with this nanoformulation (cell death values of ≈10% and ≈50%, respectively) at a total drug concentration of 0.5 µg, in the case of Taxotere^®^, a similar antiproliferative effect was detected (cell death values of ≈40%) [152]. It should be mentioned that in this study, the higher overall anticancer effect of micelles could be also related to drug combination (cisplatin plus docetaxel), as the combination of the free agents was not tested, and it cannot be concluded if the targeting strategy was effective or not. Moreover, as micelles tend to rapidly dissociate when administered, the advantage of simultaneous delivery of both drugs in tumor cells may not occur.

Active targeted polymeric micelles have also been evaluated in TNBC. PEGylated poly (D, L-lactide) micelles targeted to neuropilin-1 receptors via CK3 peptide conjugation have been developed to selectively deliver paclitaxel in tumor cells. This formulation was efficiently internalized by triple-negative cancer cells overexpressing neuropilin-1 receptors (MDA-MB-231 cells), showing a 4.4-fold higher uptake than free paclitaxel and an around 2.2-fold higher internalization compared with non-decorated micelles, indicating that neuropilin-1 targeting is a good strategy to obtain a selective location of the antineoplastic drugs in TNBC. In vivo studies in mice confirmed the tumor accumulation of CK3-decorated micelles. As a consequence, a higher selective antitumor effect was also detected in vitro and in vivo [153]. Polymeric micelles targeting luteinizing hormone-releasing hormone (LHRH) receptors were also effective to obtain a selective PTX accumulation at the tumors. This formulation consists of poly (ethylene glycol)-dendritic cholic acid micelles decorated with a peptide-binding LHRH receptor and loaded with paclitaxel. In vitro studies in TNBC cells (MDA-MB-231 and MDA-MB-435) demonstrated a higher internalization and cytotoxicity of decorated paclitaxel micelles. In vivo studies even reported that this formulation was more effective than Taxol^®^ (conventional paclitaxel formulation) [154].

Apart from paclitaxel, active targeted micelles loaded with other antineoplastics such as docetaxel or doxorubicin have also been designed for TNBC therapy. For example, polyethylene glycol micelles functionalized with folic acid, as TNBC cells tend to overexpress folate receptor-α, and loaded with docetaxel also demonstrated a selective tumor accumulation. While folic acid-decorated micelles demonstrated a higher internalization and cytotoxic effect than non-decorated ones in TNBC cells overexpressing folate receptors (4T1 cells), in cells lacking these receptors (CHO cells), both micelles exhibited a similar effect. The anticancer activity was also confirmed in vivo in xenograft mice models [155]. Folic acid and dextran-retinoid acid-decorated magnetic micelles containing doxorubicin also showed a slightly higher cytotoxic effect compared to non-targeted systems in MDA-MB-468 cells [156]; cells that also tend to overexpress this target [157]. However, in this case, the improvement in the anticancer effect was not pronounced.

Finally, poly(ethylene oxide)-poly(ε-caprolactone) micelles were also decorated with the P18-4 peptide, a ligand that targets breast cancer cells, and evaluated in TNBC. In this case, decorated micelles also demonstrated a higher tumor accumulation. Although a drug was not incorporated, this study indicates that these P18-4-functionalized micelles could be a good strategy for obtaining selective chemotherapy [158].

### 6.2. Dendrimers

Dendrimers consist of branched repeating units of a polymeric structure with a larger number of exposed cationic, anionic, or neutral groups that allow the incorporation of both lipophilic and/or hydrophilic drugs [159]. They have been exploited as nanocarriers of antineoplastic drugs such as paclitaxel, docetaxel, doxorubicin, and cisplatin [160]. Currently, several dendrimer-based formulations are approved for cell transfection or diagnostic purposes. Several formulations are also under clinical trials in therapeutics, including a formulation encapsulating docetaxel (DEP^®^-docetaxel) in patients with different tumors such as gastrointestinal and urological malignancies [161].

In HER-2-positive breast cancer models, trastuzumab-grafted poly(amidoamine) dendrimers encapsulating docetaxel showed a higher cytotoxic effect than non-grafted dendrimers or conventional docetaxel (Taxotere^®^) in MDA-MB-453 cells (they overexpress HER-2 receptors), with IC_50_ values of 56.18 ng/mL, 201 ng/mL, and >250 ng/mL, respectively. However, in cells lacking HER-2 receptors (MDA-MB-231), both dendrimers showed a similar efficacy (IC_50_ values of 149.5 ng/mL and 163.4 ng/mL of trastuzumab-grafted and non-grafted docetaxel dendrimers, respectively). A higher in vivo efficacy of trastuzumab-docetaxel dendrimers was also appreciated in MDA-MB-453 xenograft tumors models. These results are related to the higher internalization of targeted dendrimers in HER-2-overexpressing cells [162]. A higher internalization in HER-2-positive breast cancer cells (SKBR-3) was also appreciated in trastuzumab-grafted dendrimers containing neratinib, a tyrosine kinase inhibitor, as an anticancer drug. However, in this case, a higher cytotoxic effect was not detected. Although trastuzumab-neratinib dendrimers showed a higher cell proliferation inhibition (≈67%,) than neratinib dendrimers (≈64%) and free neratinib (≈61%), statistical differences were not achieved [163]. Kretzmann and coworkers also demonstrated that targeting α_v_β-family integrins, which are overexpressed on the surface and vasculature of many cancers, is a good strategy to obtain a selective accumulation of breast cancer cells. These authors developed propargyl polyamido amine (PAMAM) dendrimers functionalized with the cyclic RGD peptide. These nanosystems were efficiently internalized by MCF-7-derived tumors developed in mice [164].

In TNBC, dendrimers containing doxorubicin and grafted with angiopep-2 (it binds to low-density lipoprotein-receptor-related proteins that are overexpressed in these types of carcinomas) showed a higher internalization in 4T1 cells than non-grafted dendrimers and a higher tumor growth inhibition. Interestingly, the incorporation of these dendrimers on the surface of gelatin nanoparticles increases their antitumor effect. While angiopep-2-doxorubicin dendrimers exhibited a tumor growth inhibition of around 53%, the angiopep-2-doxorubicin dendrimers gelatin nanoparticles showed a tumor growth inhibition of around 74% [165]. Despite these promising results, this formulation is too complex, and its clinical translation is very difficult, especially those aspects related to large-scale manufacturing. Finally, an active targeted nanoformulation based on self-assembly dendrimers of polyethylene glycol derivates containing paclitaxel and grafted with a cyclic peptide binding CD13 receptors that are overexpressed in the tumor vasculature has been designed. Although this formulation exhibited a lower cytotoxic effect in MDA-MB-231 cells compared to Taxol^®^, in vivo, in xenograft tumors derived from this line and developed in mice, targeted formulation exhibited a higher tumor growth inhibition (≈82%) than non-targeted nanoformulation (≈56%) or Taxol^®^ (≈38%) [166]. The difference between the in vitro and in vivo activity is related to the fact that in tumor cell lines, active targeting is not achieved as this peptide improves the access of the formulation from the bloodstream to the tumor. Moreover, with dendrimers, paclitaxel is released slowly compared to Taxol^®^ and not all of the drug is available to exert the anticancer effect from the beginning.

### 6.3. Polymeric Nanoparticles

In the case of polymeric nanospheres, their industrial manufacturing is even more tricky than liposomes or micelles, as most currently elaboration techniques involve the use of solvents that cannot be used for large-scale productions or methods that do not guarantee reproducibility of the batches at the industrial level (e.g., uniform particle size and size distribution). This can explain why most of the formulations that have reached the clinic are liposomes. Notwithstanding, several polymeric nanoparticles have reached the clinic. Livatag^®^ consisting of non-active targeted nanoparticles of polyalkylcyanoacrylate, cyclodextrin, and poloxamer encapsulating doxorubicin is under phase III clinical trial for the treatment of hepatocarcinoma. Interestingly, there are two functionalized formulations under clinical research: (i) BIND-014, which consists of pegylated poly(lactic-co-glycolic acid) (PLGA) nanoparticles functionalized with prostate-specific membrane antigen and loaded with docetaxel and that is being evaluated in patients with prostate cancer, non-small lung cell cancer and cervical cancer [167,168]; and (ii) CALAA-01, which consists of cyclodextrin-containing pegylated PLGA nanoparticles functionalized with transferrin and that contains small-interfering RNA (siRNA) targeting the M2 subunit of ribonucleotide reductase [169] and that has been evaluated in patients with melanoma [170]. To the best of our knowledge, no active targeted polymeric nanoparticles have been evaluated in breast cancer patients. Nevertheless, many of them have been designed and tested using preclinical models of both HER-2-positive tumors and TNBC.

PLGA is the most used polymer to elaborate polymeric nanoparticles, probably due to its high biocompatibility and biodegradability. In the organism, PLGA undergoes hydrolysis producing lactic acid and glycolic acid (their monomers). It is approved by the FDA and EMA for various biomedical applications, including drug delivery, as several controlled drug delivery formulations elaborated with this polymer are currently approved [171,172]. In fact, the two polymeric nanoformulations that are currently under clinical research for cancer disease (BIND-014 and CALAA-01) are PLGA nanoparticles. In this context, numerous active targeted nanomedicines containing antineoplastics and elaborated with PLGA have been designed.

In HER-2-positive breast tumors, most of the nanoparticles are functionalized with trastuzumab. PLGA nanoparticles decorated with this monoclonal antibody and loaded with docetaxel were demonstrated to be effective in BT-474 cancer cells. While trastuzumab-decorated nanoparticles exhibited a significantly higher cytotoxic effect with an IC_50_ < 50 ng, free docetaxel and non-decorated nanoparticles showed similar antiproliferative activity, with an IC_50_ > 200 ng/mL [173]. These differences were attributed to HER-2 receptor targeting. Docetaxel-loaded hybrid nanoparticles prepared with PLGA and lipids and decorated with trastuzumab also showed a higher antiproliferative effect compared to the free drug in these HER-2-positive breast cancer cells. However, non-decorated nanoparticles exhibited a similar effect to free docetaxel [80]. Interestingly, Duan et al. demonstrated that the incorporation of an antigen-binding fragment (fab’) of trastuzumab on the surface of curcumin-loaded PLGA nanoparticles was more effective than the addition of the hole monoclonal antibody. In this case, both trastuzumab and fab’ coated nanoparticles showed a higher antiproliferative effect in BT-474 breast cancer cells than free curcumin and non-coated curcumin-loaded nanoparticles. When administered at doses of 24 µm, while free curcumin and non-coated nanoparticles showed a cell death of around 40 and 50%, respectively, nanoparticles coated with trastuzumab and fab’ produced a cell proliferation inhibition of around 70 and 80%, respectively. Moreover, a higher in vivo tumor accumulation was also appreciated in fab’-functionalized nanoparticles [174]. Finally, trastuzumab-coated PLGA nanoparticles loaded with paclitaxel demonstrated a higher antiproliferative activity in these cells compared to free paclitaxel but not to non-coated PLGA nanoparticles. In this study, the administration of paclitaxel as a free drug at 10 µm did not exert any cytotoxic effect in BT474 cells. However, the administration of both trastuzumab-coated and non-coated paclitaxel-loaded nanoparticles at the same paclitaxel concentration (10 µm) showed a cell proliferation inhibition of around 35% [175].

In TNBC, several targets have been exploited such as the CD44 receptor, a cell adhesion membrane glycoprotein overexpressed on many cancer types including triple-negative breast cancer [176], or EGFR-1 receptors. Hyaluronic acid is a CD44 receptor ligand, and, for this reason, it is grafted on the surface of polymeric nanosystems to obtain their selective localization in these cancer cells.

For example, PLGA nanoparticles coated with hyaluronic acid and loaded with paclitaxel showed a higher anticancer activity than non-coated nanoparticles in MDA-MB-231 cells. While non-coated formulations showed an IC_50_ value of around 5 nm, hyaluronic acid-decorated nanoparticles had an IC_50_ of around 3 nm. The IC_50_ of free paclitaxel was also around 5 nm. The higher cytotoxic activity of decorated formulations was related to their higher internalization as MDA-MB-231 cells overexpress CD44 receptors [177]. PLGA nanoparticles coated with hyaluronic acid and loaded with doxorubicin also demonstrated a higher antitumor activity than non-coated nanoparticles in MDA-MB-231 cells, but not in MCF-7 cells, where both formulations showed a similar cytotoxicity, as MCF-7 cells do not overexpress CD44 receptors [178]. Similar results were also obtained with formulations loaded with gambogic acid, where the higher anticancer activity of coated formulations in TNBC was also corroborated in vivo in a xenograft mice model [179].

In the case of EGFR receptors-mediated targeting, PLGA nanoparticles decorated with an aptamer binding these receptors (CL4) significantly increased the efficacy of cisplatin in TNBC. While cisplatin-loaded PLGA nanoparticles showed an IC_50_ of around 10 µm in MDA-MB-231 cells, CL4-coated cisplatin-loaded PLGA nanoparticles exhibited an IC_50_ of around 2 µm. This higher efficacy can be related to EGFR-1-mediated internalization, as in MDA-MB-231 depleted from EGFR-1, both nanoparticles exhibited a similar cytotoxic effect with IC_50_ values of around 10 µm. This was also corroborated in vivo, as a higher tumor accumulation and a higher tumor growth inhibition were detected with targeted nanoparticles, suggesting the potential EGFR-1 ligand to improve conventional chemotherapy in TNBC [180]. An improvement in paclitaxel activity was also appreciated in in vivo models of MDA-MB-468-derived tumors in mice. While free paclitaxel did not report any tumor growth inhibition, showing a similar tumor growth to the negative control, the administration of paclitaxel-loaded nanoparticles showed a tumor growth decrease of around 58%. Interestingly, nanoparticles coated with an anti-EGFR-1 ligand showed an almost complete reduction in the tumors, as the percent of tumor growth inhibition was around 96%. This is attributed to the selective accumulation of the targeted nanoparticles at the tumor mass [181]. This is also interesting in terms of toxicity, as a reduction in paclitaxel side effects could be expected.

Apart from PLGA, chitosan and polycaprolactone are other polymers used for the development of targeted nanoformulations. In HER-2-positive tumors, the incorporation of trastuzumab on the surface of chitosan nanoparticles loaded with docetaxel significantly improved the cytotoxicity of this antineoplastic. At concentrations of docetaxel of 2.5 µg/mL, non-coated nanoparticles showed a cell proliferation inhibition of around 52% while trastuzumab-coated nanoparticles showed a value of around 80% [182]. Folic acid-decorated polycaprolactone (PCL) nanoparticles loaded with methotrexate demonstrated a higher effect in HER-2-positive tumor cells. In MCF-7 cells, targeted nanoparticles showed a 3-fold lower IC_50_ than non-targeted nanoparticles due to the higher internalization mediated by folate receptors [183]. In TNBC, doxorubicin-loaded nanoparticles prepared with this polymer and coated with hyaluronic acid showed a higher tumor accumulation and tumor growth inhibition in 4T1-derived xenograft tumors developed in mice than in non-coated nanoparticles (≈65% vs. ≈96%). Higher survival rates were also appreciated [184]. Sorolla et al. demonstrated that poly(acrylic acid)-decorated poly(glycidyl methacrylate) functionalized with the EN1 peptide engineered with RGD sequences is an excellent strategy to target TNBC cells [185] and to increase the efficacy of docetaxel, as the EN1 peptide showed anticancer activity. These targeted nanoparticles were efficiently accumulated at T11-derived tumors and showed a higher tumor growth reduction than the co-administration of both non-targeted docetaxel nanoparticles plus EN1 peptide (≈27% vs. 19%) [186].

## 7. Other Nanoformulations

Cyclodextrins are cyclic oligosaccharides consisting of glucose subunits bound by α-1,4 glycosidic bonds obtained by enzymatic degradation of starch. The most common are α, β, and γ cyclodextrins, which contain 6, 7, and 8 units of glucose, respectively. They have a hydrophobic internal core and a hydrophilic surface [187,188]. In fact, they have been widely used as nanocarriers of many therapeutic agents, including antineoplastics. In breast cancer, targeted cyclodextrin nanoformulations have also been developed as a new chemotherapeutic strategy. For example, cyclodextrins conjugated with glutamine encapsulating doxorubicin have considerably improved the effect of this drug in TNBC. Breast carcinomas have a low expression of glutamine synthetase and overexpress the alanine–serine–cysteine transporter 2 to internalize this amino acid. Therefore, glutamine-conjugated systems tend to be accumulated at these tumors. This formulation showed a significantly higher tumor growth inhibition in MDA-MB-231-derived tumors. While non-conjugated cyclodextrins showed a lower effect than free doxorubicin (around 30% and 52% of tumor reduction compared with the control), the effect of glutamine conjugated cyclodextrins was much higher, with a tumor reduction of around 75% [189]. Using this in vivo model, similar results were obtained with doxorubicin-loaded cyclodextrins functionalized with mannose, as many cancers overexpress mannose receptors. Tumor growth inhibition rates of around 49% and 70% were detected in mice treated with free doxorubicin and mannose-decorated doxorubicin cyclodextrins, respectively [190]. Folic acid-decorated paclitaxel-loaded cyclodextrins also demonstrated a higher activity than free paclitaxel in 4T1-derived tumors [191].

Silica nanoparticles represent another potential nanocarrier type for the administration of antineoplastic agents, and several active targeted nanoparticles have been developed for breast cancer [192]. For example, silica nanoparticles containing doxorubicin and decorated with cyclodextrins bound to an HER-2 aptamer showed a higher cytotoxic effect in HER-2 overexpressing breast cancer cells (SKBR3 cells) than non-decorated particles. While free doxorubicin (at a concentration of 3.6 µg/mL) and non-decorated nanoparticles showed a high cell viability of around 90% and 80%, respectively, HER-2-targeted cyclodextrins showed a significantly lower cell survival of around 40% [193]. Silica nanoparticles coated with folic acid also increased the cytotoxicity of doxorubicin in MDA-MB-231 cells, as demonstrated by the reduction in IC_50_ values (3.21, 2.56, and 1.50 µg/mL of free doxorubicin, non-targeted, and folic acid-targeted nanoparticles, respectively). This was also confirmed in vivo in MDA-MB-231-derived tumors [194]. Finally, silica nanoparticles loaded with epirubicin and targeted to mucin 1 receptors have been designed. However, although a higher uptake of these targeted nanoparticles has been detected in MCF-7 cells, a higher antiproliferative effect was not detected, as both non-targeted and targeted nanoparticles exhibited a similar cell proliferation inhibition. In fact, this effect was slightly lower than free epirubicin, which can be related to the controlled drug release of epirubicin from silica nanoparticles [195].

Finally, polyrotaxanes, formed by the interaction of cyclodextrins and polymers, such as poly(ethylene glycol), functionalized with folic acid significantly increased the anticancer activity of doxorubicin. In vitro, folic acid-decorated doxorubicin-loaded polyrotaxanes showed a higher cytotoxic effect in MCF-7 cells than non-decorated nanoparticles and free paclitaxel. This was also corroborated in vivo, where a tumor growth inhibition of around 25%, 62.5%, and 75% was detected with free doxorubicin, non-decorated, and folic acid-decorated nanoparticles, respectively [196].

## 8. Preclinical to Clinical Translation: Challenges and Future Perspectives

Despite all of the advantages of using nanosystems as carriers of antineoplastic drugs for the treatment of cancer in general and breast cancer in particular, the translation from preclinical research to effective clinical outcomes has presented many difficulties and has met with limited success with many formulations, especially of active targeted nanomedicines (Figure 5). It is well known that nanomedicines lose their original features when they are in contact with biological fluids, interfering with their distribution, toxicity, and efficacy [197]. One of the factors that significantly affect the behavior of nanosystems when they are administered in the human body is the formation of the protein corona, a shell of biological molecules (mainly proteins but also lipids, sugars, and metabolites) formed on the nanoparticle surface [198]. This affects the stability of the nanoformulations and interferes with their biodistribution. In the case of active targeted systems, this protein corona can mask the targeting moiety, hindering their accumulation in tumors and affecting their efficacy [199]. Moreover, this can increase their immunorecognition by the components of the mononuclear phagocyte system and their blood clearance. To prevent this, many formulations have been coated with polyethylene glycol molecules (i.e., have been “pegylated”) [200]. However, the incorporation of polyethylene glycol on the particle surface can result in a complement activation—pseudo allergy, producing treatment failure due to the loss of efficacy or the appearance of toxicities [201]. For example, this has been observed in approved doxorubicin-loaded liposomes. Moreover, these pegylated liposomes of doxorubicin (Doxil^®^) have another adverse effect that does not appear in conventional doxorubicin formulations: palmar-plantar erythrodysthesia. Due to the incorporation of polyethylene glycol, these liposomes showed a very small particle size that accumulated in the skin triggering this reaction [12]. In fact, the toxicity of nanomedicines, unpredictable in many cases, is one of the main problems for their authorization. Although it is true that nanomedicines tend to accumulate in tumors, especially active targeted formulations, due to their small particle size and capacity to interact with biological structures, they can access other regions of the organism, accumulate, and penetrate inside cells, leading to significant adverse effects. Precisely for this reason, nanomedicines that use materials present in the body (such as albumin or phospholipids in liposomes) as nanocarriers are more readily approved, as they are expected to have fewer toxicity problems.

Tumor heterogeneity, in terms of the level of angiogenesis, tumor fibrosis, degree of epithelial–mesenchymal transition, and receptor expression, also represents a handicap that may prevent the clinical anticancer activity of the nanosystems. First, all this conditions the accumulation of nanomedicines by the EPR effect, and, consequently, their efficacy. In fact, one of the main reasons that many nanosystems are not effective for the treatment of solid tumors in humans is due to their poor penetration into tumors. Wilhem et al. reported that less than 1% of the injected dose reaches tumors [202]. Secondly, these tumor characteristics can interfere in the internalization of the active targeted nanoformulations mediated by receptors overexpressed on the cell surface [203,204]. In fact, many clinical trials have demonstrated that the efficacy of these nanomedicines is correlated to the expression levels of the target. 

Another difficulty involved in the preclinical to clinical translation of nanomedicines is related to their design, as all the physicochemical properties of the nanoformulations (particle size, zeta potential, morphology, and carrier composition, among others) determine the interaction of nanoformulations with the biological membranes and cells, and, consequently, their efficacy. This interaction at the nanoscale is different in animals than in humans, so it is difficult to extrapolate the preclinical data to humans [205,206]. In the case of active targeted formulations, the engineering strategy to incorporate the targeting moiety is also crucial, especially in antibody–drug conjugates as the antibody is bound to the drug and this binding may alter the drug effect in terms of efficacy [207]. In fact, several antibody–drug conjugates were ineffective as the drug was not efficiently released or was released maintaining remnants of the linker or amino acids, and this interferes in their mechanisms of action.

Another important challenge is the controlled and reproducible industrial manufacturing of nanomedicines under good manufacturing practice (GMP) conditions. Since subtle changes during the production of nanomedicines can affect their physicochemical properties and, therefore, their efficacy and toxicity, the control of all elaboration parameters is especially critical in large-scale manufacturing. The challenge of industrial manufacturing is also related to the type of nanocarriers, as some techniques are easier to optimize industrially [12]. For example, this occurs with liposomes, with several optimized techniques for large-scale production. This can explain, at least in part, why many of the nanomedicines that have reached the clinic (not only in cancer but also in other pathologies such as infectious diseases) are liposomes. This also happens with antibody–drug conjugates. Also related to their manufacture, nanomedicines must be sterile for human use (considering that they are administered parenterally), and the sterilization technique can alter the performance of nanocarriers [208], especially those that contain biologicals such as monoclonal antibodies or polymers [209]. For example, γ-irradiation may affect drug release from polymeric nanoparticles [210]. Among all sterilization techniques, filtration could be useful, as it does not affect nanomedicine characteristics. However, it cannot be used with all formulations [211].

Despite all their advantages over conventional chemotherapy, the number of nanomedicines that reach the clinic is still very low compared with the number of nanomedicines that have been developed at the preclinical level, and much lower are those that are finally approved, especially in the case of active targeted nanomedicines, due to all these challenges related to a lack of higher clinical efficacy, toxicity problems, and complex manufacturing. In fact, major efforts are currently underway to develop preclinical models that better mimic clinical conditions in humans and allow obtaining more “realistic” results in terms of toxicity and efficacy, as well as new methods that simplify the large-scale production of nanomedicines and allow the elaboration of more reproducible formulations, as is the case of microfluidics [211]. It could be expected, that in the following decades, these challenges could be, at least in part, overcome. In fact, the use of active targeted nanomedicines in cancer, in general, and breast cancer, in particular, as a strategy to optimize and improve anti-tumor treatments by using a more personalized therapy, is on the rise, especially in the case of antibody–drug conjugates. Only in the last 4 years, seven antibody–drug conjugates have been approved by the FDA and/or EMA for the treatment of several neoplasms, two of them for breast cancer. Many other formulations are also under clinical trials, with more expected approvals over the next few years.

## 9. Conclusions

The use of nanocarriers selectively targeted to tumor cells is a good strategy to optimize conventional chemotherapy, reducing its toxicity and increasing its effectiveness. In both HER-2-positive and triple-negative breast tumors, many active targeted nanoformulations have been designed with this purpose, and a total of 16 active targeted nanomedicines have reached the clinic. Different targets have been used for the design of these active targeted nanoformulations. In the case of HER-2-positive breast cancer, the most widely used target is undoubtedly HER-2 receptors, due to their high overexpression in this type of tumor and the good results obtained with therapies targeted to these receptors. In the case of triple-negative breast cancer, numerous targets have been evaluated, including Trop-2 receptors, zinc transporter LIV-1, PTK-7, NMB glycoproteins, and ephrin receptors. Among them, nanosystems targeting Trop-2 receptors, zinc transporter LIV-1, and PTK7 have shown great promise, especially the first, with an already approved formulation. Finally, among all nanoplatforms, antibody–drug conjugates are particularly promising active targeted nanoformulations, with three approved formulations and the other seven under clinical investigation for breast cancer. 

## Figures and Tables

**Table 1:** Nanomedicines that are currently approved for breast cancer treatment.

Brand Name	Anticancer Drug	Type of Formulation	Indications	Approval Year
Doxil^®^ (Caelyx^®^)	Doxorubicin	Pegylated liposomes	Breast cancer	FDA (1995)
			Ovarian Cancer	EMA (1996)
			AIDS-related Kaposi’s sarcoma	
Myocet^®^	Doxorubicin	Non-pegylated liposomes	Metastatic breast cancer	EMA (2000)
Lipusu^®^	Paclitaxel	Non-pegylated liposomes	Non-small cell lung cancer	China (2006)
			Ovarian cancer	
			Breast cancer	
Abraxane^®^	Paclitaxel	Albumin nanoparticles	Advanced non-small cell lung cancer	FDA (2005)
			Metastatic breast cancer	
			Metastatic pancreatic cancer	EMA (2008)
Genexol^®^	Paclitaxel	Polymeric micelles	Non-small cell lung cancer	South Korea (2007)
			Breast cancer	
Nanoxel^®^	Paclitaxel	Polymeric micelles	Metastatic breast cancer	India (n.a)
			Non-small cell lung carcinoma Kaposi’s sarcoma	
Bevetex^®^	Paclitaxel	Polymeric-lipidic nanoparticles	Ovarian cancer	India (n.a)
			Breast cancer	
			Bladder cancer	
Kadcyla^®^ (Trastuzumab- emtansine)	DM1 (maytansinoid)	Antibody–drug conjugate	HER-2-positive breast cancer	FDA (2013)
				EMA (2013)
Enhertu^®^ (Trastuzumab-Deruxtecan)	Deruxtecan (camptothecin)	Antibody–drug conjugate	Metastatic HER-2-positive breast cancer	FDA (2019)
				EMA (2021)
Trodelvy^®^ (Sacituzumab-Govitecan)	SN-38 (camptothecin)	Antibody–drug conjugate	Metastatic TNBC	FDA (2020)
				EMA (2021)

FDA: Food and Drug Agency, EMA: European Medicine Agency, n.a: non-available.

**Table 2:** Nanomedicines under clinical research in breast cancer.

	Formulation	Drug	Target	Ligand	Indication	Clinical Phase (NCT Number)
Antibody-drug conjugates	Trastuzumab-duocarmycin	Duocarmycin	HER-2	Trastuzumab (anti-HER-2 monoclonal antibody)	HER-2-positive breast cancer	Phase III (NCT03262935
	Hertuzumab-MMAE (RC48-ADC)	MMAE (auristatin)	HER-2	Hertuzumab (anti-HER-2 monoclonal antibody)	HER-2-positive breast cancer	Phase I (NCT02881190/NCT02881138)
	HT19-MMAF (XMT-1522)	MMAF (auristatin)	HER-2	HT19 (anti-HER-2 monoclonal antibody)	HER-2-positive breast cancer	Phase I (NCT02952729)
	Glembatumumab-Vedotin	MMAE (auristatin)	NMB glycoprotein	Glembatumumab (anti NMB glycoprotein monoclonal antibody)	TNBC	Phase II (NCT01997333) ^a^
	Ladiratuzumab-vedotin	MMAE (auristatin)	LIV-1	Ladiratuzumab (anti LIV-1 monoclonal antibody)	Metastatic breast cancer	Phase I/II (NCT01969643/NCT03310957)
	Cofetuzumab-pelidotin (PF-06647020)	Aur001 (auristatin)	PTK7	Cofetuzumab (anti-PTK7 monoclonal antibody)	TNBC	Phase I (NCT03243331/NCT02222922)
	PF-06647263	Calicheamicin	Ephrin receptor-4	Anti Ephrin receptor -4 monoclonal antibody	TNBC	Phase I (NCT02078752)
Albumin nanoparticles	Nab-rapamycin	Rapamycin	gP 60 receptors	Albumin	Solid tumors	Phase I (NCT02646319)
			SPARC proteins			
Liposomes	MM-302 (anti-HER-2 pegylated liposomes)	Doxorubicin	HER-2	Single-chain fraction of an anti-HER-2 monoclonal antibody	HER-2-positive breast cancer	Phase I-III (NCT01304797, NCT02213744)
	C225-ILS-Dox	Doxorubicin	EGFR	Antigen-binding fragment of cetuximab	TNBC	Phase II (NCT02833766) ^b^
	MM310	Docetaxel pro-drug	Ephrin A2	Anti-ephrin A2 monoclonal antibody	Solid tumors ^c^	Phase I (NCT03076372)

^a^ Discontinued; ^b^ prematurely terminated; ^c^ including triple-negative breast cancer, cervical cancer, endometrial cancer, pancreatic cancer, prostate cancer, urothelial cancer, gastric cancer, and small cell lung cancer among others. HER-2 epidermal growth factor receptor, PTK7: protein tyrosine kinase 7, TNBC: triple-negative breast cancer.
